# Positive Psychological Micro-Interventions to Improve the Work–Family Interface: Use Your Resources and Count Your Blessings

**DOI:** 10.3389/fpsyg.2020.00275

**Published:** 2020-02-28

**Authors:** Maria C. W. Peeters, Elianne F. van Steenbergen, Jan Fekke Ybema

**Affiliations:** ^1^Department of Social, Health and Organizational Psychology, Utrecht University, Utrecht, Netherlands; ^2^Industrial Engineering and Innovation Sciences, Eindhoven University of Technology, Eindhoven, Netherlands; ^3^Dutch Authority for the Financial Markets (AFM), Amsterdam, Netherlands

**Keywords:** cognitive appraisal, work–family conflict, work–family enrichment, positive psychology, micro-interventions

## Abstract

The present study is designed to test the effectiveness of two positive psychological micro-interventions (“use your resources” and “count your blessings”) aimed at improving the combination of work and family roles. Based on the Transactional Model of Stress (TMS), the Conservation of Resources (COR) Theory and the Work-Home Resources (WH-R) Model, it was expected that the interventions would result in a more positive cognitive appraisal of combining both roles as well as in less work-to-family and family-to-work conflict and more work-to-family and family-to-work enrichment. The hypotheses were tested in a field experiment with three conditions and three measurement waves. In total, 218 working mothers participated in the study. The “use your resources” intervention appeared effective in sorting positive effects on the work–family outcome variables. Participating in the “count your blessing” micro-intervention did not result in a better (appraisal of the) combination of work and family roles. Moreover, for generating positive effects it was important that the participants performed the exercises on a regular basis: the more days women performed the exercise, the stronger the effects. The implications of our findings for future interventions to improve work–family role combining are discussed.

## Introduction

Over the past decades, the traditional gendered division of work and family roles has eroded and made place for dual-earner couples in which both partners combine paid work with family roles. Combining multiple roles is typically assumed to be difficult and stressful and a large body of research has shown that work can indeed negatively interfere with fulfilling family roles and vice versa (Allen et al., 2000; Van Steenbergen et al., 2008; Amstad et al., 2011).

However, on the positive side of role combining, work and family roles have also the potential to enrich one another (Greenhaus and Powell, 2006). Numerous studies have shown that the extent to which individuals experience role conflict and role enrichment has far reaching consequences for their health, performance and wellbeing both at work and at home (e.g., McNall et al., 2010; Reichl et al., 2014; Zhang et al., 2018) and for their relationships with partner and children (e.g., Van Steenbergen et al., 2014; Fellows et al., 2016). Therefore, scholars are increasingly trying to design interventions that help individuals to better combine their multiple roles, such as training supervisors to support the family domain (Hammer et al., 2011), offering new ways of working (Demerouti et al., 2014) or implementing mindfulness-based training at the workplace (Kiburz et al., 2017). Up till now, most interventions in this area are top down initiatives from for example the Human Resources Department and employees may or may not sign up for it. Less attention has been paid to the design of interventions that focus on actions that employees themselves can initiate on a daily basis to improve the combination of work and family roles. The present research tries to fill this gap by developing and testing two positive micro-interventions that are specifically designed to provide employees with techniques that are supposed to facilitate the combination of different roles. By designing micro-interventions that are easy to apply we expect employees to be more inclined to use them in their daily lives. The purpose of both interventions is to influence the cognitive appraisal of combining multiple roles. Based on the Transactional Model of Stress (TMS; Lazarus and Folkman, 1984), The Conservation of Resources (COR) Model (Hobfoll, 1989) and the Work-Home Resources (WH-R) Model (Ten Brummelhuis and Bakker, 2012) we assume that a more positive appraisal of the combination of roles will encourage employees to perceive less role conflict and more role enrichment.

## Theoretical Background

### The Cognitive Appraisal of Combining Work and Family Roles

The TMS, developed by Lazarus and Folkman (1984), conceptualizes the occurrence of stress as “psychologically mediated.” The central construct in this model is the individual’s cognitive appraisal of a situation, which intervenes between the objective occurrence of a certain situation and the reaction of the individual to that specific situation. This cognitive appraisal consists of a primary and secondary appraisal. In *primary appraisal* the individual evaluates how stressful a certain situation is. If this situation is categorized as stressful, the appraisal can be characterized in three ways: (1) harm, which refers to the fact that harm is already experienced, (2) threat, which is harm that is anticipated, and (3) challenge, which is the potential for mastery or gain. A harm appraisal refers to harm in the past. Threat and challenge appraisals refer to ongoing or upcoming situations.

*Secondary appraisal* occurs almost at the same time, and the basic question here is “Can I cope?” When an individual assesses his/her available resources as sufficient to deal with the situation, the situation is likely to be perceived as challenging. This creates positive thoughts about the situation and more positive emotions, like enthusiasm, and motivation (Lazarus and Folkman, 1984). If one’s resources seem insufficient to cope, the individual will perceive the situation as more threatening. He or she will feel anxious, emotionally overwhelmed, and will keep worrying about the situation (Van Steenbergen et al., 2008). Primary and secondary appraisals converge to determine whether the combination of multiple roles is regarded as significant for well-being, and if so, whether it is primarily threatening or challenging (Folkman et al., 1986).

### The Influence of Positive Micro-Interventions on the Cognitive Appraisal of Role Combining

The present study aims to investigate whether two positive micro-interventions can influence how individuals cognitively appraise the combination of their work and family roles. The micro-interventions are originally designed by Seligman et al. (2005) and are called “three good things in life” and “use signature resources in a new way.” The interventions were designed from a positive psychological perspective to reduce the symptoms of depression by influencing thoughts positively. The interventions are called micro-interventions because they can be performed in a relatively short time period without intensive contact with a trainer or counselor. In “three good things in life” participants were asked to write down three good things that happened that day. The participants were also asked to explain each good thing that happened. “Use signature strengths in a new way” implied that participants had to find out their top five character strengths and use one of these in a new and different way every day for one week (Seligman et al., 2005). Both interventions reduced the symptoms of depression and increased happiness, even six months after the intervention. Because of these promising short and long-term effects we wanted to test the suitability of similar micro-interventions in the context of role combining. To this purpose we adapted both interventions slightly and applied them to the context of the present study. To distinguish our micro-interventions from the ones by Seligman and colleagues we labeled them as “use your resources” (based on use signature strengths in a new way) and “count your blessings” (based on three good things in life).

In the “use your resources” intervention participants are encouraged to use their personal resources to improve their functioning at work, at home, or both. In line with the TMS, we argue that being aware of one’s personal resources makes a stressor easier to handle and as a result people will appraise it more as a challenge and less as a treat. In addition, becoming more aware of one’s personal resources and applying them, will also improve secondary appraisal (“can I cope?”) of role combination. This intervention would then lead to a more positive experience of the combination of work and family roles.

In the “count your blessings” intervention participants are encouraged to think about the positive things that happened on a particular day. We argue that this counting of blessings makes people more aware of their good things in life and makes them focus more on the positive side. This mindset will lead people to experience stressors less as a threat and more as a challenge. In turn this will lead to more positive thoughts, emotions and actions, and as a result a better combination of work and family life.

Van Steenbergen et al. (2008) already examined this phenomenon. In a field experiment, participants were provided with information that supported either a role expansion perspective or a scarcity perspective on the combination of work and family roles. Their findings showed that working mothers experienced their combination of work and family roles as more positive when they were provided with information that supported a role expansion perspective as compared to information that supported a scarcity perspective.

These insights indicate that, on a practical level, there are opportunities to develop intervention programs that can ameliorate the stress caused by the combination of both work and family roles. This means that potentially, interventions can be used to impact upon people’s cognitive appraisals of combining work and family roles. Building on the TMS and previous research, we expect that:

H1: Compared to participants in the control condition, participants in the intervention conditions will appraise role combining (a) less as a threat and more as a challenge (primary appraisal) and (b) will have a more beneficial secondary appraisal, both directly after the intervention (T1) and three weeks later (T2).

### The Influence of Positive Micro-Interventions on Conflict and Enrichment

By cognitively construing the task of combining work and family roles as a threat or as a challenge, over time, the extent to which individuals experience conflict and/or enrichment as a result of combining roles, will be affected. A conflict between work and family can be defined as: “a type of role conflict that arises when joint role pressures from the work and family domains are experienced as incompatible in some respect, as a result of which participation in one role is made more difficult by virtue of participation in the other role” (Greenhaus and Beutell, 1985, p. 77). These conflicts can arise in both the work-to-family direction and the family-to-work direction (Byron, 2005). Research in this tradition is predominantly influenced by the *scarcity perspective* on the fulfillment of multiple roles and human energy (see Marks, 1977). The basic assumption here is that available time and energy resources are limited and that the fulfillment of multiple roles is likely to result in a depletion of these scarce resources. However, according to Marks’ (1977) *role-expansion approach* individuals can also experience enrichment between roles. This approach posits that human energy is abundant and expendable, and that roles can also positively affect one another.

Work–family enrichment is defined as the individual’s experience that participation in one role makes it easier to fulfill the requirements of another role (Greenhaus and Powell, 2006).

Work–family enrichment also occurs in two directions, namely work-to-family enrichment and family-to-work enrichment.

In line with research of Van Steenbergen et al. (2007), we argue that different types of conflict and enrichment need to be distinguished in order to better understand the different ways in which role-combining is experienced. In the present study we chose to focus on the energy/strain type as well as on the psychological type of conflict and enrichment. We omitted behavioral and time-based conflict and enrichment to keep the survey as short as possible, and because we expect that our interventions will tap more into the former two aspects of the work–family interface. *Energy-based enrichment* takes place when energy obtained in one role makes it easier to fulfill the requirements of another role. *Strain-based conflict* means that strain produced in one role can make it difficult to fulfill the requirements of another role (Carlson et al., 2000). *Psychological conflict* refers to the situation that psychological preoccupation with one role prevents one from becoming engaged in another role (Carlson and Frone, 2003). Finally, *psychological enrichment* occurs when an individual is able to put matters associated with one role into perspective by virtue of another role, which makes it easier to fulfill the requirements of the first role (Van Steenbergen et al., 2007).

An important aim of the present study is to investigate if the two positive psychological micro-interventions are powerful enough to achieve that – on the longer run – individuals will appraise the work–family combination less conflicting and more enriching. The COR theory (Hobfoll, 1989) as well as the WH-R model (Ten Brummelhuis and Bakker, 2012) provide arguments for an expected positive answer to this question. A basic tenet of the COR theory is that people strive to retain, protect and build resources that they value. COR theory describes two main processes: The first is a *loss spiral*, in which stress develops and resources further deplete, and the other is a *gain spiral*, in which resources accumulate. Those with greater resources are less vulnerable to perceive stress, and additionally they are more capable of future resource gain (Hobfoll, 1989, 2002). The W-HR model uses the loss and gain process of the COR theory to build a theoretical argument regarding the interface between work and home. Conflict between work and family occurs when stress in a particular domain depletes resources so that these resources are no longer available for functioning in the other domain. Enrichment occurs when resources from one domain lead to the development of resources in another domain which subsequently facilitates outcomes in the other domain. To summarize, as the names already imply, resources play a central role in both COR theory and W-HR model. Perceiving a loss of resources in either domain will result in perceiving the combination of roles as conflicting and perceiving resources gain in either domain will lead to a perception of role combining as enriching.

The preservation and use of resources is also at the core of the two micro-interventions that we are going to test in the present study. In the “use your resources” intervention participants are motivated to use the resources they already have at their disposal whereas in the “count your blessing intervention” participants are trained to value the resources they already have. We expect that both using as well as valuing resources will help employees to perceive the combination of work and family role after a few weeks (at T2) as less conflicting and more enriching. So, based on these theoretical insights we expect that:

Compared to the control condition, participants in the intervention conditions will experience at T2:

H2a: lower WF conflict (strain-based, psychological)
H2b: higher WF enrichment (energy-based, psychological)
H2c: lower FW conflict (strain-based, psychological)
H2d: higher FW enrichment (energy-based, psychological)

## Materials and Methods

### Design and Procedure

This study is a field experiment with three conditions and three measurement waves. Participants in the intervention conditions participated in an intervention week of seven days. Before the intervention, participants filled out the baseline questionnaire (T0), directly after the intervention week the first follow-up measurement (T1), and three weeks later the second follow-up measurement (T2) to examine the effects of the intervention in the somewhat longer run. Consistent with previous research (Van Steenbergen et al., 2008), we focus on working mothers of young children because the combination of work and family roles is a very salient issue for this group of employees. Criteria for inclusion were: (1) having at least one child aged five years or younger, (2) cohabiting with partner, and (3) both the participant and the partner had to work at least 24 h a week.

Participants were recruited by eight students who collaborated with the researchers. Students recruited in day-care centers, schools, and in shopping areas in and around the city of Utrecht in the Netherlands. In addition, both the students and the primary researchers recruited couples via email in their own networks and used a snowball technique to recruit more couples. The recruitment posters and emails stated the aim of the study, its voluntary nature, what was expected from participants, and that a voucher worth twenty euros ($26) could be won when participating in the whole study. Participants could sign up via an email address that was created for the study. Upon signing up, they were informed about the dates before which the three surveys had to be completed.

Participants were randomly categorized into the three conditions: (1) “use your resources,” (2) “count your blessings,” and (3) control. To connect the data over time, participants were asked at each measurement moment to fill in the same unique personal code. Anonymity was ensured as this code was created by the participants themselves and was unknown to the researchers. Participants received all instructions and invitations to complete the surveys via email, and received reminder text messages for the surveys. After the first questionnaire (T0), participants were informed via email to which condition they were assigned.

The two interventions were designed in a highly similar fashion. Participants received a text (about 1.5 pages) that (a) gave information about the method (count your blessings or use your resources) and its theoretical roots in positive psychology. Then (b) a fictitious quote was given of a woman with two young children who described why she decided to participate in a training learning this method. Subsequently (c), instructions for the diary exercises were given, and participants were informed that they would receive an email with the URL to their diary every day at 7 p.m. Then (d) another fictitious quote was given by the same women describing how the method had helped her. Finally, (e) participants were instructed to perform the exercise as good as possible, and the importance was emphasized of performing the exercise every day. More specific information about the diary exercises is given below. The complete intervention texts are available upon request.

#### Diary Exercise Use Your Resources

The day before the diary exercises started, participants were instructed to reflect on their personal resources. We provided some examples of what this could be such as being assertive, energetic or optimistic, or having a good sense of humor, good organizational skills or financial resources. We then stated that it was step one to be aware of these resources, and step two to deploy them to manage their work, home life, or the combination of work and home more effectively or more pleasantly. Participants were instructed to write down their top 3 of personal resources. Each of the seven days, they had to deploy one of these resources in a conscious way to improve their work, their home life or the combination of these two. Each day, participants could choose in what area they wanted to implement the resource. As they could choose one of their three personal resources, some resources could be chosen more than once. Participants were instructed to apply the personal resource each day in a different way. Every day, participants were asked to write down on what personal resource they focused that day and in which area they deployed it. They also had to answer the question: “What effect did it have on you?”

#### Diary Exercise Count Your Blessings

The “count your blessings” diary exercise consisted of daily counting your blessings in one’s work, home life or concerning the combination of work and home life. We provided some examples of what this could be such as having enjoyable or pleasant experiences or nice social encounters. Participants were instructed to, at least one time a day (preferably at a set time), think about the positive things that happened that day. Every day, participants were instructed to write down two blessings of the day in their diary. They could choose in which area (work, home, or the combination) they were counting their two blessings. For both blessings they had to answer the question: “Why was this experience positive?”

### Participants

In total, 360 participants were recruited and randomly assigned to one of the three conditions. Of these participants, 240 completed all three surveys (overall response rate 67%). The response rate for the “use your resources” condition was 67%, for the “count your blessings” condition 73% and for the control condition 61%. Out of the 240 participants, 14 participants were removed because they did not fit the inclusion criteria and eight because they did not answer the manipulation check correctly. This resulted in a sample of *N* = 218 participants: *N* = 63 in the “use your resources” condition, *N* = 79 in the “count your blessings” condition, and *N* = 72 in the control condition. Age of the participants ranged from 22 to 46 years (*M* = 35, SD = 4.1) and almost all participants were highly educated (92%). There were no significant differences between the three conditions on the background variables, which were number of children, age of the participant, level of education, number of hours worked, and number of hours worked by the partner.

### Measures

All measures were either existing Dutch measures or back translated from English.

Items were answered on 5-point rating scales (1 = fully disagree, 5 = fully agree) unless stated otherwise.

*Cognitive appraisals* were assessed with measures developed by Kessler (1998), which we adapted to the situation of combining work and home roles. A 5-item scale assessed the extent to which participants appraised role-combining as a *threat* (e.g., “The combining of my work and home life is frightening to me,” αT0 = 0.81, αT1 = 0.87, αT2 = 0.85). A six-item scale assessed the extent to which participants appraised role-combining as a *challenge*, e.g., “The combining of my work and home life enables me to learn more about myself,” (αT0 = 0.81, αT1 = 0.84, αT2 = 0.87). Secondary appraisal was assessed with a five-item scale e.g., “I have influence on the way in which I combine my work and home life.” From this measure, we excluded one item (“I can change things in the way I combine my work and home life”) because this resulted in more acceptable reliabilities, namely: αT0 = 0.67, αT1 = 0.66, αT2 = 0.67 (instead of αT0 = 0.67, αT1 = 0.62, αT2 = 0.63). All items were answered on seven-point scales (1 = fully disagree, 7 = fully agree).

#### Work–Family Conflict (WFC)

*Strain-based WFC* was assessed with the three-item scale developed by Carlson et al. (2000), e.g., “Due to all the pressures at work, sometimes when I get home I am too stressed to do the things I enjoy,” αT0 = 0.74, αT1 = 0.81, αT2 = 0.84.

*Psychological WFC* was also assessed with a 3-item scale (Carlson and Frone, 2003), e.g., “When I am at home, I often think about things I need to accomplish at work” (αT0 = 0.83, αT1 = 0.85, αT2 = 0.86).

#### Work–Family Enrichment (WFE)

*Energy-based WFE* and *Psychological WFE* were measured with two three-item scales (Van Steenbergen et al., 2007). Respectively, sample items and reliabilities were “When I get home from work I often feel emotionally recharged, enabling me to make a better contribution at home” (αT0 = 0.83, αT1 = 0.89, αT2 = 0.86) and “Because of my work, I am better able to put home-related matters into perspective” (αT0 = 0.80, αT1 = 0.82, αT2 = 0.86).

#### Family–Work Conflict (FWC)

*Strain-based FWC* was measured with a three-item scale developed by Carlson et al. (2000) and *psychological FWC* was measured with a three-item scale developed by Carlson and Frone (2003). Respectively, sample items and reliabilities were: “Tension and anxiety from my home life often weakens my ability to do my job” (αT0 = 0.89, αT1 = 0.91, αT2 = 0.89) and “When I am at work, I often think about things I need to accomplish at home” (αT0 = 0.81, αT1 = 0.84, αT2 = 0.87).

#### Family–Work Enrichment (FWE)

*Energy-based FWE* and *Psychological FWE* were measured with the three-item scales developed by Van Steenbergen et al. (2007). Respectively, sample items and reliabilities were “Because I relax and regain my energy at home, I can better focus on performing my work” (αT0 = 0.83, αT1 = 0.83, αT2 = 0.89), and “Because of my home life, I am more able to put work-related matters into perspective” (αT0 = 0.82, αT1 = 0.87, αT2 = 0.86).

#### Manipulation Check and Level of Participation

In the second questionnaire (T1), we included for the participants of the intervention conditions an item that served as a manipulation check, namely “What exercise did you perform during the previous week?” Participants could choose between “count your blessings” and “use your resources.” As stated earlier, eight participants gave the wrong answer and we removed them from our sample. In addition, participants were asked: “How many days have you managed to actually perform the exercises seriously?” (0–7 days). Unfortunately, in the “count your blessings” condition, the average number of days (*M* = 5.3, SD = 1.7) was higher than in the “use your resources” condition (*M* = 4.2, SD = 2.0), *F*(1, 144) = 12.2; *p* < 0.001. To account for this difference in the implementation of the intervention, in all analyses not only the effects of the manipulation were examined, but also the additional effects of the number of days a participant performed the exercises.

### Statistical Analyses

The data were analyzed using hierarchical linear regression (OLS) in SPSS 22 for Windows. To test the hypotheses, in the regression of the dependent variable at T1 (appraisal) or T2 (appraisal, conflict, enrichment), the dependent variable at baseline (T0) was entered in the first step. In the next step, two dummy variables concerning the manipulation were entered in the regression to test whether both interventions differed from the control condition. This step tested the main effects of the manipulation. In the third step, a variable indicating the number of days the participant had done the exercises in either of the intervention conditions was entered in the regression. In the final step the interaction between the type of intervention (“count your blessings” or “use your resources”) and the number of participated days was entered in the regression. Participants in the control condition scored 0 on both dummy variables and on both number of days variables (main effect and interaction variable), and constitute the baseline for testing each effect in the regression. Participants in the “count your blessings” condition scored 1 on the “count your blessings” dummy and 0 to 7 on the number of days variable (main effect), and 0 on both other variables. Participants in the “use your resources” condition scored 1 on the “use your resources” dummy and 0 to 7 on both number of days variables (main effect and interaction effect), and 0 on the dummy variable for the other condition. The final regression gives full information on the influence of the manipulation and the number of days the participants did their exercises in both intervention conditions. It should be noted that predictors were not centered to the mean in order to keep the control condition as the baseline for testing each effect in the regression. To avoid faulty interpretations of the regression weights, we present the results of the regression hierarchically rather than the final regression equation.

## Results

### Descriptive Statistics and Correlations at Baseline

Table 1 presents the descriptive statistics and correlations between appraisals, work–family conflict and enrichment, family–work conflict and enrichment at baseline.

**TABLE 1:** Descriptive statistics and correlations of the dependent variables at baseline (*N* = 218).

		Range	*M*	SD	1	2	3	4	5	6	7	8	9	10	11
1	Threat appraisal	1–7	3.09	1.19	1.00										
2	Challenge appraisal	1–7	5.23	0.88	−0.52*	1.00									
3	Secondary appraisal	1–7	5.06	0.95	−0.44*	0.33*	1.00								
4	Work–family conflict strain	1–5	2.57	0.90	0.53*	−0.46*	−0.38*	1.00							
5	Work–family conflict psychological	1–5	3.30	0.98	0.33*	−0.20*	–0.04	0.24*	1.00						
6	Work–family enrichment energy	1–5	2.91	0.89	−0.27*	0.33*	0.21*	−0.56*	−0.16*	1.00					
7	Work–family enrichment psychological	1–5	3.47	0.92	–0.04	0.16*	0.07	−0.15*	–0.00	0.37*	1.00				
8	Family–work conflict strain	1–5	1.85	0.85	0.35*	−0.25*	−0.34*	0.21*	–0.02	–0.06	0.01	1.00			
9	Family–work conflict psychological	1–5	2.47	0.92	0.26*	–0.12	–0.13	0.05	0.02	–0.02	0.18*	0.48*	1.00		
10	Family–work enrichment energy	1–5	3.70	0.81	−0.20*	0.35*	0.13	−0.17*	–0.02	0.20*	0.14*	−0.22*	–0.08	1.00	
11	Family–work enrichment psychological	1–5	4.04	0.77	–0.12	0.29*	0.17*	−0.27*	–0.12	0.16*	0.25*	–0.04	0.07	0.22*	1.00

*p < 0.05.

Table 1 shows that threat appraisal correlated strongly negatively with challenge and secondary appraisal, whereas challenge and secondary appraisal were moderately positively related. Moreover, strain-based work–family conflict correlated strongly positively with threat appraisal, and strongly negatively with energy-based work–family enrichment. In general, the correlations between strain/energy-based and psychological conflict or enrichment were only moderate. A multivariate analysis of variance showed that there was neither an overall difference at baseline, *F*(22, 412) = 0.87, ns, nor significant differences between conditions for any of the individual variables at baseline, *F*(2, 215) < 2.37, ns. Thus, randomization worked as anticipated.

### Threat, Challenge, and Secondary Appraisals

Hypothesis 1 stated that both interventions would contribute to lower threat and higher challenge appraisals of combining work and family in both the short term and the long term. Moreover, secondary appraisal – i.e., perceived coping opportunities – was predicted to improve following both interventions. To test this, hierarchical regressions of threat, challenge, and secondary appraisals directly after the intervention (T1) and three weeks later (T2) were carried out to examine the effects of the interventions and the number of days the participants carried out the exercises. The results are presented in Table 2.

**TABLE 2:** Regression of threat, challenge and secondary appraisals directly after the intervention (T1) and 3 weeks later (T2).

		Threat T1		Threat T2		Challenge T1		Challenge T2		Secondary appraisal T1		Secondary appraisal T2	
							
Step	Predictor	B	SE B	B	SE B	B	SE B	B	SE B	B	SE B	B	SE B
1	Constant	0.73*	0.17	0.85*	0.16	2.61*	0.30	2.64*	0.31	2.08*	0.27	2.40*	0.27
	Threat T0	0.70*	0.05	0.64*	0.05								
	Challenge T0					0.51*	0.06	0.54*	0.06				
	Secondary appraisal T0									0.61*	0.05	0.55*	0.05
2	Dummy count blessings	–0.03	0.15	–0.03	0.14	0.14	0.12	0.13	0.12	0.04	0.12	0.09	0.12
	Dummy use resources	–0.18	0.15	−0.38*	0.14	0.15	0.13	0.19	0.13	0.21	0.12	0.14	0.13
3	Days participated	–0.06	0.04	–0.00	0.04	0.07*	0.03	0.08*	0.03	0.01	0.03	0.08*	0.03
4	Interaction Intervention*Days	–0.09	0.08	–0.04	0.08	0.21*	0.06	0.19*	0.07	0.20*	0.06	0.16*	0.07

	*R*^2^	0.46*		0.45*		0.33*		0.34*		0.43*		0.37*	

*N* = 218; *p < 0.05.

In the regression of threat appraisals, the first step showed that threat appraisal at baseline contributed strongly to the regression of threat appraisal at both T1 and T2. In step 2, the main effects of the intervention did not contribute significantly to the regression of threat appraisal at T1 [*F*(2, 214) < 1.0; ns] but – partly in line with the hypothesis – it did contribute significantly to the regression of threat appraisal at T2 [*F*(2, 214) = 4.4; *p* < 0.05]. As can be seen in Table 2, only the “use your resources” intervention reduced threat appraisals in the long run compared to the control condition. Neither the number of participated days nor the interaction between the type of intervention and the number of participated days contributed significantly to the regression of threat appraisals at T1 or T2.

**FIGURE 1:**
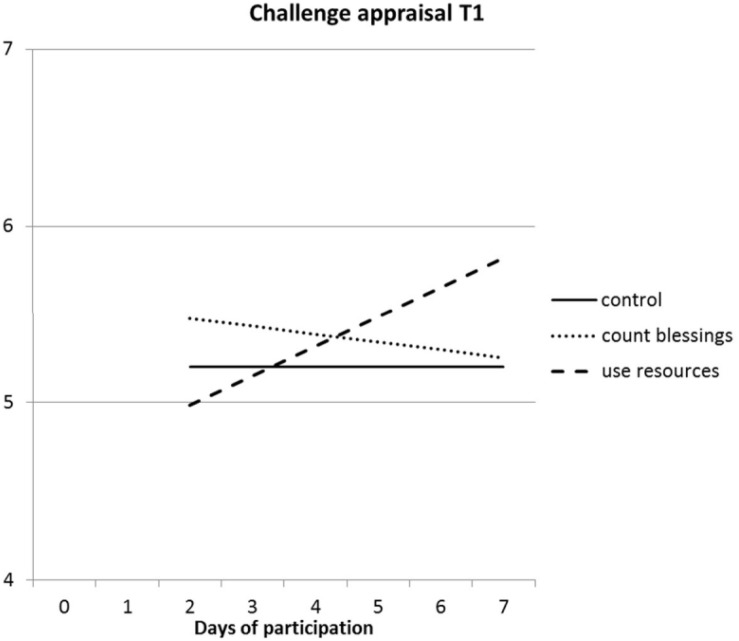
Interaction effect between type of intervention and number of days participated in the intervention on challenge appraisal at T1.

The regressions of challenge appraisals showed that challenge appraisal at baseline contributed strongly to the regression of challenge appraisals at both T1 and T2. Contrary to hypothesis 1, the main effects of the intervention did not contribute significantly to the regressions [*F*(2, 214) < 1.2; ns]. Nevertheless, both the number of participated days and the interaction between the type of intervention and the number of participated days contributed significantly to the regression of challenge appraisal at T1 and at T2. Figure 1 depicts the regression lines for challenge appraisal at T1 on the number of participated days in both interventions groups. The graphic for challenge appraisal at T2 was almost identical.

It can be seen that for participants in the “use your resources” condition, the more days they did their exercises, the more they regarded combining work and family as a challenge. This was not true for participants in the “count your blessings” condition. The simple slope for the number of participated days was significant in the “use your resources” condition in both regressions of challenge appraisals at T1 and T2 (*p* < 0.001).

In the regressions of the secondary appraisal, secondary appraisal at baseline contributed strongly to the regression of secondary appraisals at both T1 and T2. Contrary to the hypothesis, the main effects of the intervention did not contribute significantly to the regressions [*F*(2, 214) < 1.7; ns]. Nevertheless, the number of participated days contributed to the regression of secondary appraisal three weeks after the intervention (T2), and the interaction between the type of intervention and the number of participated days contributed significantly to both the regression of secondary appraisal directly after the intervention (T1) and three weeks later (T2). The graphics for secondary appraisals were highly similar to those presented in Figure 1 for challenge appraisals at T1. For participants in the “use your resources” condition, the more days they did their exercises, the more coping opportunities they experienced for combining work and family, both in the short and the long term. This was not true for participants in the “count your blessings” condition. The simple slope for the number of participated days was significant and positive in the “use your resources” condition in both regressions (T1, *p* < 0.05; T2, *p* < 0.001), but negative in the “count your blessings” condition directly after the intervention (T1, *p* < 0.05).

### Work–Family Conflict

Hypothesis 2a stated that the interventions would reduce both strain-based and psychological work–family conflict in the long run. Hierarchical regressions of T2 WF conflict are presented in Table 3.

**TABLE 3:** Regression of strain-based and psychological work–family conflict and energy-based and psychological work–family enrichment.

		WF conflict strain T2		WF conflict psychological T2		WF enrichment energy T2		WF enrichment psychological T2	
					
Step	Predictor	B	SE B	B	SE B	B	SE B	B	SE B
1	Constant	0.50	0.14	0.75*	0.16	1.29*	0.16	1.99*	0.20
	WF conflict strain T0	0.73*	0.05						
	WF conflict psychological T0			0.71*	0.05				
	WF enrichment energy T0					0.66*	0.05		
	WF enrichment psychological T0							0.49*	0.06
2	Dummy count blessings	–0.10	0.11	0.01	0.11	0.08	0.11	–0.04	0.12
	Dummy use resources	–0.22	0.12	–0.17	0.11	0.11	0.12	0.09	0.13
3	Days participated	–0.04	0.03	−0.08*	0.03	0.05	0.03	0.04	0.03
4	Interaction Intervention*Days	–0.08	0.06	–0.06	0.06	0.22*	0.06	0.15*	0.07

	*R*^2^	0.49*		0.55*		0.47*		0.28*	

*N* = 218; *p < 0.05.

As can be seen, strain-based and psychological work–family conflict were highly stable: they were strongly predicted by these variables at baseline. No main effects of the interventions were found [*F*(2, 214) < 1.7; ns] but for psychological WF conflict, the number of participated days contributed significantly to the regression. This means that as participants did their exercises on more days, they experienced lower psychological WF conflict. However, the interaction between type of intervention and participated days was not significant in both regressions.

### Work–Family Enrichment

Hypothesis 2b stated that the interventions would increase both energy-based and psychological work–family enrichment in the long run. Hierarchical regressions of T2 WF enrichment are presented in Table 3.

**FIGURE 2:**
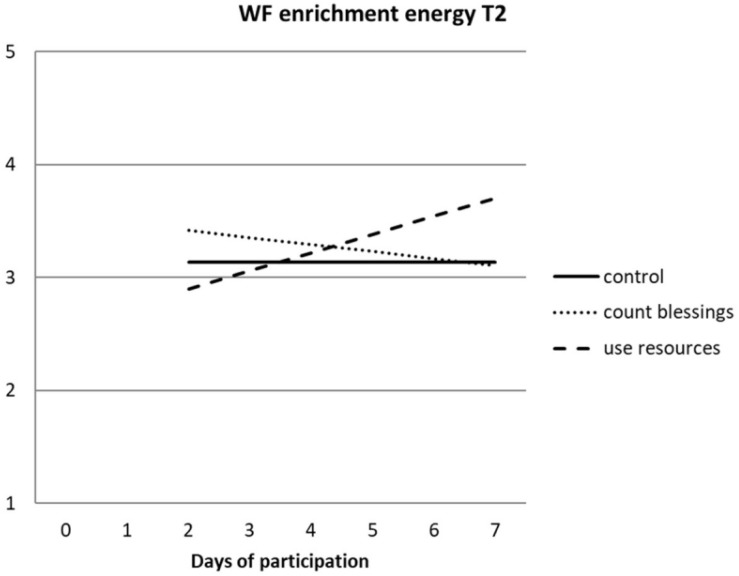
Interaction effect between type of intervention and number of days participated in the intervention on the energy dimension of work–family enrichment at T2.

As can be seen, energy-based work–family enrichment was more stable than psychological work–family enrichment. Again, no main effects of the interventions were found [*F*(2, 214) < 1.0; ns]. However, the interaction between type of intervention and participated days was significant in both regressions. Apparently, the relationship between the number of participated days and WF enrichment was different for participants in both conditions. Figure 2 shows the graphics for energy-based WF enrichment at T2. The graphics for psychological work–family enrichment was highly similar to those presented in Figure 2.

Both energy-based and psychological WF enrichment increased as participants in the “use your resources” condition did their exercises on more days, but not in the “count your blessings” condition. In both regressions, the simple slope for the number of participated days was significant for the “use your resources” intervention (energy-based, *p* < 0.001; psychological, *p* < 0.05), but not for the “count your blessings” intervention.

### Family–Work Conflict

Hypothesis 2c stated that the interventions would reduce both strain-based and psychological family–work conflict in the long run. Hierarchical regressions of T2 FW conflict are presented in Table 4.

**TABLE 4:** Regression of family–work conflict, family–work enrichment.

		FW conflict strain T2		FW conflict psychological T2		FW enrichment energy T2		FW enrichment psychological T2	
					
Step	Predictor	B	SE B	B	SE B	B	SE B	B	SE B
1	Constant	0.81*	0.11	0.74*	0.13	2.29*	0.20	2.37*	0.23
	FW conflict strain T0	0.57*	0.06						
	FW conflict psychological T0			0.69*	0.05				
	FW enrichment energy T0					0.44*	0.05		
	FW enrichment psychological T0							0.42*	0.06
2	Dummy count blessings	–0.21	0.11	0.06	0.11	0.12	0.10	0.14	0.10
	Dummy use resources	–0.10	0.12	0.08	0.12	0.24*	0.10	0.22*	0.11
3	Days participated	–0.01	0.03	–0.02	0.03	–0.00	0.03	0.05	0.03
4	Interaction Intervention*Days	–0.00	0.06	0.00	0.06	0.10	0.05	0.13*	0.05

	*R*^2^	0.34*		0.47*		0.28*		0.25*	

*N* = 218; *p < 0.05.

As can be seen, strain-based and psychological family–work conflict were highly stable: they were strongly predicted by these variables at baseline. No main effects of the interventions were found [*F*(2, 214) < 1.9; ns]. The number of participated days nor the interaction with type of intervention contributed to either regressions of FW conflict.

### Family–Work Enrichment

**FIGURE 3:**
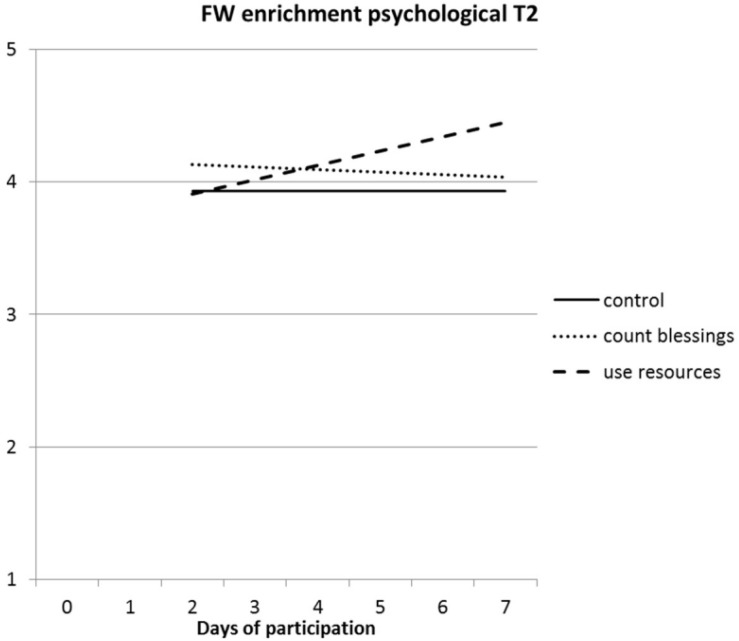
Interaction effect between type of intervention and number of days participated in the intervention on the psychological dimension of family-to-work enrichment at T2.

Hypothesis 2d stated that the interventions would increase both energy-based and psychological family–work enrichment in the long run. Hierarchical regressions of T2 FW enrichment are presented in Table 4. Both energy-based and psychological family–work enrichment were moderately stable. There was a marginally significant effect of the interventions for energy-based FW enrichment [*F*(2, 214) = 2.6; *p* < 0.10], but not for psychological FW enrichment [*F*(2, 214) = 2.1; *p* > 0.10]. For both energy-based and psychological FW enrichment, the “use your resources” intervention seemed to increase FW enrichment compared to the control condition. The number of participated days did not contribute to the regression of energy-based or psychological FW enrichment. The interaction between type of intervention and participated days was significant for psychological FW enrichment, but not for energy-based FW enrichment. The regression lines for psychological FW enrichment are presented in Figure 3.

It can be seen that psychological FW enrichment increases as participants in the “use your resources” condition did their exercises on more days, but not in the “count your blessings” condition. In this regression, the simple slope for the number of participated days was significant for the “use your resources” intervention (*p* < 0.001).

## Discussion

The goal of the present study was to develop and test two positive micro-interventions (i.e., “use your resources” and “count your blessings”) which were based on the TMS (Lazarus and Folkman, 1984) and on insights derived from the positive psychology movement (Seligman et al., 2005) and aimed to influence the combining of work and family roles. In a field experiment among working mothers we examined whether participants of the micro-interventions showed an increase in challenge appraisal, a decrease in threat appraisal and an increase in perceived coping options after the intervention, compared to participants of the control group. In addition, we examined whether participants indicated to experience less work–family and family work-conflict, and more work–family and family–work enrichment.

In general, the results revealed that the daily exercise “use your resources” was successful in improving (the appraisal of) the combination of work and family roles. Participating in the “count your blessing” micro-intervention did not result in a better appraisal of combing work and family roles. Moreover, we also detected that for generating positive effects it was very important that the participants performed the exercises on a regular basis.

We found that the more days women performed the exercise, the stronger the effect. Below we discuss the study findings and implications in more detail.

### “Use Your Resources” Intervention and (the Cognitive Appraisal of) Role Combining

Results showed that the more days women did this exercise, the more they appraised combining work and family as a challenge and the more coping opportunities (secondary appraisal) in dealing with role combination they experienced, both directly after the intervention and a few weeks later. Participating in this intervention also appeared to reduce threat appraisals, but only after a few weeks and it did not matter how many days one complied with the instructions. Apparently it takes longer before the “use your resources” micro-intervention generates its effects in terms of reduced threat appraisal. Generally, our findings support previous studies alluding that cognitive appraisal processes play an important role in combining work and family roles (Edwards and Rothbard, 2000; Green et al., 2011). More specifically, the results are in line with the study of Van Steenbergen et al. (2008) which also showed that it is possible to exert influence on the way in which individuals appraise their own combination of work and family roles. As such, our results substantiate the idea that the appraisal of the work–family interface can be influenced to some extent. Hence, the theoretical framework of the TMS appeared a good choice for demonstrating this.

Not only did the cognitive appraisal of the work–family interface change as a result of participating in this intervention, also the experience of the work–family interface itself changed in the expected direction. We examined several indicators of the work–family interface (conflict and enrichment in both directions and on an energy and psychological level) and found a rather straightforward pattern of results. Both energy-based and psychological WF enrichment increased as the participants in the “use your resources” condition did their exercises more frequently. With regard to energy-based FW enrichment the results also showed that this increased for participants in the “use your resources” conditions. For the psychological dimension of FW enrichment, this was dependent of the number of days they participated in the exercises: The more days they did the exercise, the stronger the effect. Participating in the “use your resources” intervention did not seem to be effective in reducing conflict. We distinguished between four types of role conflict (strain-based WF and FW conflict and psychological WF and FW conflict) and none of the types of role conflict showed significant differences between the participants in the three conditions. Apparently, the positive psychological “use your resources” intervention is effective in increasing positive states but less suitable for reducing negative states. This underlines the belief of many scholars in the area of positive psychology that positive psychology interventions are not explicitly designed to treat negative states: they are designed to build positive qualities.

Hence, if a positive intervention succeeds to reduce negative outcomes, this should be considered as an unexpected positive side effect (Sin and Lyubomirsky, 2009; Meyers et al., 2013). This in in line with a study by Ouweneel et al. (2014) who found that some positive psychological interventions seemed to foster positive emotions and academic engagement, but did not decrease negative emotions. One could also speculate that this has something to do with the nature of the sample. Since our sample was a non-clinical sample consisting of working women who were not on sick leave because of depression, burnout or otherwise, negative emotional states due to role combination may have been less prevalent than positive states, leading to a so-called “floor effect” for these negative states. In general, Sin and Lyubomirsky (2009) state that in clinical samples positive interventions are more likely to affect negative states, though in non-clinical samples this is probably not the case.

Our results can also be interpreted in line with a review of Gilbert et al. (2018) which shows promising evidence that personal resources can be built through simple interventions hereby contributing to many aspects of workers’ performance and well-being. Although our intervention was not aimed at *building* resources but at *using* resources, it both refers to the same underlying premise that personal resources are beneficial for employee wellbeing and functioning and are as such worthwhile to invest in.

In general, the results of the “use your resources” intervention are in line with a review of 15 studies on the effects of positive psychological interventions in organizations (Meyers et al., 2013). The review showed that these type of interventions consistently enhanced employee well-being and, to a lesser degree, performance. However, as there were no studies included in the review that were specifically developed to test the added value of positive psychology interventions for the work–family interface, we should be a bit careful in drawing this conclusion. The results of the present study first need to be replicated. Surprisingly, to the best of our knowledge, ever since the promising results of the study of Van Steenbergen et al. (2008), no other researchers have taken up the challenge to apply positive psychology interventions to the work–family domain. We hope this study will stimulate new researchers to do so because we think that positive psychology (micro)-interventions have a lot to offer to the work–family domain.

### “Count Your Blessings” Intervention and (the Cognitive Appraisal of) Role Combining

Unexpectedly, participating in the “count your blessings” intervention did not change the appraisal of the combination of the work–family interface, nor did it influence the experience of the work–family interface itself, neither in the short run nor in the longer run. One possible explanation for this unexpected result could be that behavioral positive interventions (like using your resources) work better than cognitive interventions (like counting your blessings). A similar effect was found by Ouweneel et al. (2014). They found that the cognitive intervention “thoughts of gratitude” was less effective than the behavioral intervention “acts of kindness” in generating positive emotions and academic engagement. Behavioral interventions might evoke more immediate positive feedback than cognitive interventions because it is mostly about overt behavior that can be seen and rewarded by important others like the partner, children or colleagues. However, as other studies do report promising effects of the “three good things exercise” (on which our count your blessings exercise is based) (e.g., Bono et al. (2013) this explanation certainly warrants more research.

### Exercise Frequency as a Moderator

It is well-known from the literature that a number of factors moderate the effects of positive psychology interventions. Voluntary participation seems to be the most crucial one. Two major meta-analyses (Sin and Lyubomirsky, 2009; Bolier et al., 2013) showed that self-selected volunteers derive greater well-being benefits (average *r* = 0.35) than did participants who were assigned (average *r* = 0.20). Our results add to this knowledge that the frequency of doing exercises also is an important factor for generating positive effects of the intervention. For the “use your resources” intervention we found stronger positive effects when the participants performed the exercise on more days. This aligns with results of a study of Lyubomirsky et al. (2011) who found that effort is associated with the magnitude of resource gains. Participants experienced larger gains when they expect the exercise to be useful *and* when they completed it repeatedly and attentively. Unfortunately attrition rates are often high, in studies of both online volunteers and workers (e.g., Page and Vella-Brodrick, 2013). So we can carefully conclude that employees who complete interventions as meant, tend to experience more resource gains (Meyers et al., 2013), but still little is known about those who drop out.

### Limitations and Implications for Future Research and Practice

Our study is not without limitations. First, due to our sampling technique we have a selective sample which makes it difficult to generalize the results of his study to the total population of working mothers. More studies should try to replicate the present findings in order to more fully understand what works for whom. Second, we compared two intervention groups with a control group that did not receive an intervention. Future studies could include a second control group that receives another kind of intervention of which no effect is expected. This could probably address the potential influence of certain experimenter demands, like getting attention, on the dependent variables. Another limitation refers to the duration of the intervention. An intervention of seven days is a relatively short period for trying to structurally change appraisals and behaviors of individuals. Performing the exercises for a longer time period can possibly lead to more effectiveness of the interventions. Thus, future research could investigate whether a positive psychological intervention that entails a longer period of exercises is more effective. This would be in line with a study on habit formation, which showed that changes in lifestyle became automatic over a period ranging from 18 to 254 days, with a median of 66 days (Lally et al., 2010). As far as we know, no such study has yet been carried out for more psychological interventions such as using your strengths or counting your blessings, but it seems likely that for such psychological habits to form also a longer period of practice would be beneficial. As our results indicated at the same time, that the more frequently the exercises were performed during the seven days, the stronger the effect was on the outcome, it would also be worthwhile to examine the optimal frequency of exercises in future studies, in addition to examining the optimal duration of the intervention.

Also, in our study we focused on conflict and enrichment as outcome measures. Future research could examine if positive psychological micro-interventions also have implications for other outcomes, preferably more objective, behavioral outcomes such as positive organizational behavior or positive social behavior in the work and family domains (as rated by others). In a related vein, new research could also consider the working of certain moderator variables like self-regulatory strength. It can be expected that individuals with good self-regulatory capabilities are better able to adhere to the exercises and to adapt their behavior in accordance with the changing appraisal of the situation. Finally, future research could explore the possibilities to let participants perform both interventions simultaneously. Since the intervention “count your blessings” mainly tries to influence the primary appraisal and the intervention “use your resources” mainly tries to influence the secondary appraisal, letting participants perform both interventions could enhance the results. This aligns with the so-called shotgun approach (Sin and Lyubomirsky, 2009) in which individuals practice multiple positive psychological interventions. This may be more effective than engaging in only one activity (e.g., Seligman et al., 2005).

Regarding theoretical advancements, the present study shows that the TMS not only provides a promising framework to study some of the mechanisms through which individuals’ cognitions about their combination of work and family roles influences their experiences in combining both roles, but also points to specific ways to intervene in this process. In addition, although positive psychological interventions have been used in general contexts to increase happiness (for an overview, see Lyubomirsky, 2007) as well as in work contexts to increase work engagement (for an overview, see Schaufeli and Salanova, 2010), the present study is to our knowledge, the first study that applies these insights in the context of combing multiple roles, hereby adding specifically to this literature. Moreover, the COR theory and W-HR model prove valuable in better understanding *why* the “use your strengths” intervention works. The COR theory posits that gain spirals occur when one’s resources lead to the acquisition of even more resources. The W-HR model explains more specifically how resources in one domain (e.g., work) can lead to the acquisition of resources in another domain (e.g., home), hence resulting in better outcomes in the “receiving domain.” Take an employee who decides to use her strength “kindness to others” more at work. At work, she for example offers to help a co-workers out who is very busy. This act of kindness may be reciprocated by co-workers at times when she herself is very busy, which increases her perceived social support at work. Because of this positive experience, this worker also starts to offer help to other working parents, which is reciprocated in such a way that she is building a network of parents in the neighborhood who help each other out. This example illustrates how skills learned at work enrich the home domain.

Since our study showed that one micro-intervention (use your resources) is effective in improving employees’ appraisal of their work-life balance, it has immediate and fairly easy to implement practical implications. This seems important because, preferably, interventions need to be integrated into the busy days of individuals for whom “time” is an important factor to reckon with (Meyers et al., 2013). Because the intervention is short, simple, and self-guided, there is also little in the way of costs or drawbacks for organizations. Thus, this intervention seems like a potentially useful component of workplace work–family initiatives. We recommend organizations to organize brief workshops in which the technique is explained and participants can practice under supervision of a qualified coach. After that, employees can use the strategy “use your resources” on their own initiative and on a daily basis in order to boost their work–family balance. So for employees who want to improve their work–family balance, the simplest advice we can give is: “Use your resources!”

## Data Availability Statement

The datasets generated for this study are available on request to the corresponding author.

## Ethics Statement

Ethical review and approval was not required for the study on human participants in accordance with the local legislation and institutional requirements that were valid at the time of data collection. The patients/participants provided their written informed consent to participate in this study.

## Author Contributions

MP and ES were responsible for the conception and design of the study and organized the database. MP wrote the first draft of the manuscript. ES and JY wrote sections of the manuscript. JY performed the statistical analyses. All authors read and approved the final submitted version.

## Conflict of Interest

The authors declare that the research was conducted in the absence of any commercial or financial relationships that could be construed as a potential conflict of interest. The handling Editor declared a shared affiliation, though no other collaboration, with one of the authors MP at time of review.
